# Roles of Chemical Complexity and Evolutionary Theory in Some Hepatic and Intestinal Enzymatic Systems in Chemical Reproducibility and Clinical Efficiency of Herbal Derivatives

**DOI:** 10.1155/2014/732045

**Published:** 2014-04-06

**Authors:** Francesco Di Pierro

**Affiliations:** Scientific Department, Velleja Research, Viale Lunigiana 23, 20125, Milano, Italy

## Abstract

Despite the great marketing success, most physicians attribute poor efficacy to herbals. This perception is due to two situations that are an integral part of the herbal topic. The first is the poor phytochemical reproducibility obtained during the production process of herbal extracts, as herbal extracts are not always standardized in the whole manufacturing process, but only in their titer. The second problem is linked to the evolution of important enzymatic systems: cytochromes and ABC proteins. They are both enzyme classes with detoxifying properties and seem to have evolved from the molecular mould provided by active plant substances. During the evolution, as still happens today, polyphenols, saponins, terpenes, and alkaloids were ingested together with food. They do not possess any nutritional value but seem to be provided with a potential pharmacological activity. Cytochromes and ABC proteins, which evolved over time to detoxify food from vegetable chemical “actives,” now seem to limit the action of herbal derivatives. The comprehension of these 2 events may explain the origin of the widespread scepticism of physicians about herbal medicine and suggests that, after correct herbal standardization, use of antagonists of cytochromes and ABC systems will make it possible to recover their pharmacological potential.

## 1. Preface

The latest report of* Feder Salus* (an association whose tasks also include the monitoring of sales volumes of food supplements in Italy) on 2012 data shows that, in spite of a severe and well-known financial crisis that has hit the country since about 2009, sales of food supplements through the “pharmacy” channel alone amount to more than 1.67 billion Euros, which corresponds to more than 111 million packs of food supplements. This value increases by a further 250 million Euros if one also includes such distribution channels as mass market and parapharmacies, with a value in packs amounting to more than 30 million pieces. In total, excluding sales on the web which are difficult to monitor, we can assess the overall turnover of food supplements at almost 2 billion Euros, corresponding to about 150 million packs sold in 2012 alone. In terms of trends over the previous year, these values account for an increase between 3.5 and 5% depending on the sales channel taken into consideration [1]. It may be assumed that the situation in other European countries is not very different, although there may be some obvious differences relatable to the average financial status and the number of inhabitants. According to the Italian legislation (number 169/2004), food supplements must be notified to the Ministry of Health prior to marketing. All notified products are listed on the website of the above ministry under “Food Supplements” [2]. We can gather from the analysis of these lists that about 65% of all notified preparations are based on herbs, that is, made up of a formula containing one or more products extracted from plants. The other 35% corresponds to symbiotic preparations, preparations based on salts/vitamins and/or amino acids. This means that there are a large number of consumers who choose to use plant-based formulations. This choice essentially reflects 4 fundamental motivations: (a) a strong attraction due to the aspects of naturalness of these preparations; (b) a strong disappointment linked to aspects of nonresolution of diseases by most of conventional medicine; (c) a strong concern about the alleged or apparent toxicity possessed by most drugs; and (d) an irrational and unscientific choice against use of synthetic chemicals which, by definition, are considered dangerous [3–5]. In spite of the large numbers characterizing the sales of plant-based food supplements and their huge diffusion, we cannot certainly deny that a large part of the medical class is decidedly sceptical [6, 7] about the “pharmacological and clinical strength” possessed by these preparations. There are some physicians, perhaps not so well prepared as they should in terms of pharmacognosy and/or pharmacology of extracts [8], who have probably forgotten the important role played by medicinal plants during the last century—from the applications in analgesia made possible by the use of opium derivatives [9], to the oncological use of yew species derivatives [10]—and consider herbal food supplements something whose clinical action is mostly due to a simple placebo effect. Another opinion widely shared by physicians is that use of botanicals is instrumental in creating dangerous interactions capable of invalidating the medical therapy they have prescribed to their patients. In fact, we know that almost one-third of consumers of herbal products choose autonomously, without seeking medical advice, to use and self-administer these preparations even in the course of an otherwise prescribed medical therapy [11]. Paradoxically, some scepticism can also be perceived, albeit partially, within that medical class that, contrary to what is claimed above, certainly has a high degree of education in the use of herbal medicines and often resorts to them. How else can we explain the title of a publication on the analysis and subsequent complaint of a herbal preparation that was found to have been adulterated with nimesulide? For example, the title of the publication “Too much effectiveness from a herbal drug” [12] would sound better as “A new case of adulteration for a herbal food supplement”. One really wonders what feeds so much scepticism. Although it is true that the issue of sophistication and/or adulteration of these products seems to affect a high percentage of herbal preparations, especially those coming from China or India [13, 14]—and this fact subtracts confidence in the sector—it must be recognized that the same also affects (with even higher percentages if we evaluate the trade carried on in certain channels like the web) the trade of some synthetic drugs, with very serious consequences [15, 16]. A lack of education in the matter, as already mentioned above, will surely contribute to the anomaly. The degree of phytotherapy competence of most physicians is certainly modest because of the lack of a proper university education. But perhaps the blame for this is largely derived from the clinician's perception that, in any case, plant-based food supplements have quite a modest efficacy. The right question to ask at this point is the reason for this low-profile perception in terms of pharmacological and clinical efficacy. In other words, why are herbals perceived as low-efficacy formulations? The answer exists but is actually complex and encompasses many different aspects of the phytotherapeutic science. In order to be able to answer this question, therefore, it will be necessary to approach each of these aspects separately.

## 2. The Issue of the Actual Reproducibility, Standardization, and Titration of Herbal Drugs

**Figure 1:**
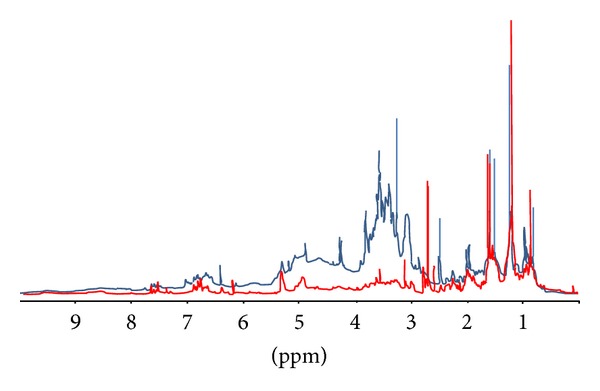
Comparative ^1^H-NMR of two* Hypericum perforatum*. Extracts; the 2 extracts have been manufactured starting from the same aerial parts, by the same solvents and have the same chemical titre (0.3% hypericin).

Scientists, no matter what scientific branch they deal with, rest the whole experimentation and the possibility of stating something resulting from the experimentation (a piece of information, a theory, etc.) on reproducibility of events. In the same way, the physician—a scientist—rests his considerations (clinical efficacy, toxicological hazards, etc.) on reproducibility of events. One of the problems that undoubtedly have had a negative influence on the aspects of the perceived efficacy of a herbal product is its difficult, albeit possible, chemical reproducibility. Two ginseng extracts, like two St. John's Wort or devil's claw extracts, obtained with the same extraction procedure and exhibiting the same titration, may be extremely different from a phytochemical point of view. And two phytochemically different preparations will also lead to different clinical and toxicological events [17]. This difference depends on titration and standardization. “Titration” of an extract generally means the numerical/percentage analysis of a substance or a type of substances that can be found in the extract. Titration is quite a modest piece of scientific information as it only tells in what percentage a certain compound or a certain fraction of compounds is found in the molecular 100% of the extract. For example, if the titration of an* Echinacea angustifolia* extract exhibits a 4% titre in echinacoside, this will only tell us that, in every 100 molecules found in the extract, 4 are echinacoside molecules [18]. This numerical evaluation is not 100% accurate as it does not take account of two parameters: the molecular weight of the substance considered and the molecular water content, being this last known to technicians as “water activity” and indicating the remaining portion of water after desiccation. For the purposes of this document, however, not taking into account of both the molecular weight of the considered substance and the weight alteration linked to the water activity may be considered negligible [19]. Obviously if echinacoside does not correspond to a real active ingredient accounting for the clinical action of the extract, this piece of information becomes almost useless and unfortunately, in many ways, even dangerously misused. For example, if a process of the same plant does not result, as expected, in a product titrated in 4% echinacoside, who or what, apart from personal ethics, can prevent the manufacturer from adding pure echinacoside to the preparation until the desired 4% is obtained? Not to speak of the problems that would arise from a further addition of echinacoside to the product, which may result in a higher value in the preparation, such as, for example, 8%. Claiming an 8% rather than 4% titration may suggest “greater activity” or a “better product”, or may be perceived by the user as a better product than that commonly prepared at 4%. As a matter of fact, if echinacoside is not a real active ingredient contributing to the ultimate action of the herbal product of which it is part, this sophistication may paradoxically have reduced the action of the product. If the molecular reason underlying the action of the botanical product had little to do with the 4% echinacoside fraction, this could mean that the “efficacious” molecules are found in the remaining nontitrated 96%. And in this case the adulteration with a further addition of echinacoside might only generate a dilution effect of this unknown, and perhaps active, fraction. This means that if the titre is not a certainly active fraction of the herbal drug, it has little, if any, clinical importance. And its value can be used in an instrumental or fraudulent manner. Another frequently used term in phytotherapy is “standardized” [20]. Unlike titration, standardization of a herbal drug is something that has a lot to do with the actual repeatability of the botanical extract and, consequently, with the scientific significance of the pharmacological and clinical results that can be obtained from its use [21]. Standardizing means normalizing all the procedures that serve to “build” the product [22], from plant sowing to the actual industrial manufacturing of the relevant herbal drug (going through all the steps, from the chemical assessment of the soil where the plant grows to the chemical-analytical characterization of the molecules present in the final extract). A standardized preparation should thus be a guarantee of an at least virtual repeatability of the molecular 100%. Two extracts obtained from the same plant, sown and harvested at the same time from the same cultivation area and from the same plant part (root, leaves, flowers, etc.), using the same solvent and so on and should be equal to each other in molecular terms if they are truly standardized. This true molecular standardization is extremely difficult to obtain from a chemical point of view because of too many random variables that may intervene throughout the process. It is true, however, that if one tries to standardize production procedures—from the chemistry of the soil where the plant will be sown to the size of the extractor used for an individual batch—herbal products should tend to quite a high degree of repeatability and be even superimposable. To date this situation—actual standardization of preparations, which thus prove to have a high phytochemical repeatability—is quite rare and obtained only by those manufacturers that really standardize the whole process. It is obvious, however, that two extracts from the same botanical species, obtained with the same solvent and exhibiting the same titration, in the absence of adulterations, but produced by two different manufacturers, will have an extremely low degree of molecular superimposition and often different repeatability degrees in terms of clinical efficacy and toxicological hazard. This clearly means that clinicians approaching herbals sooner or later should confront this problem. When using preparation A in patients, they may obtain certain benefits that they would not observe when using preparation B, although on paper the latter is identical to A (in terms of botanical species, part used for extraction, solvent, and titre claimed by the manufacturer). And all this in the hypothesis that production of A by the same manufacturer is based on an actual standardization process. Because if a manufacturer has not actually standardized the process, even 2 A products would be phytochemically different, and consequently the clinical or toxicological data resulting from their use would not be reproducible. Thinking in terms of herbal medicine production on a global scale, today it is possible to rely at best on preparations that are “standardized in their titre,” that is, having a certain degree of molecular repeatability of the titrated fraction/s [23, 24]. A certain degree of molecular repeatability can be guaranteed only by manufacturers that have really standardized the whole internal manufacturing process of a herbal drug, from the type of soil used for cultivation to extraction, desiccation, and storage methods. Consider that even today most herbals are made using harvested (not cultivated) plants, which are not the same age, come from different distributional areas and show different contents and chemical complexity right from the start, even before extraction. There is a situation, however, in which herbal drugs, standardized exclusively in their titre, are less affected by the aspects of incomplete phytochemical reproducibility. Apart from extractive preparations where the extraction solvent remains in the final preparation and exceedingly dilutes the extracted solute, where the solute reproducibility is likely to amount to nothing, and where reproducibility can only apply to the solvent used, botanical preparations from which the solvent (water, ethanol, mixtures of water and ethanol, or other solvents used in accordance with current regulations on solvents usable in the food industry) has been removed through desiccation—that is, soft, oily or dry extracts—can be divided into 2 different groups. There are extracts whose action is easily attributable to a particular chemical fraction, as is the case with boswellic acids from* Boswellia serrata* [25–27], with procyanidolic oligomers from* Crategus spp*. [28, 29] or with triterpenes selected from* Centella asiatica* [30–32]. Then there are extracts in which this information is much less, or even extremely less, apparent as is the case with* Harpagophytum procumbens* [33–36],* Valeriana officinalis* [37–39], or* Hypericum perforatum* [40–42]; for these extracts it is absolutely impossible to state with certainty that the action of the preparation can be specifically attributed to harpagoside, valerenic acids, or hypericin, the molecules in which these botanical products are titrated. It is clear from the above considerations that titre standardization may be good enough to guarantee the repeatability of a clinical datum for those preparations for which there is, with some degree of certainty, a correlation between a certain titrated molecular fraction and the measurable activity of the whole preparation. We will come back to this issue in the paragraph below. On the other hand, it seems difficult to guarantee the repeatability of a clinical result, in relation to the nonperfect repeatability of a molecular phytochemical datum, of extracts that are “only” certainly standardized in their titre and when the titrated fraction cannot be undoubtedly correlated with the action of the total derivative. The above issues linked to “titration” and “standardization” are jointly responsible, along with other problems that we will see below, for the perception of the pharmacological inadequacy of herbal drugs. Just think of the theoretical case of an extensively investigated preparation A being not only titrated (and marketed through that titre), but also actually standardized in terms of its production process, so that it proves to be repeatable in its molecular 100%. Let us also imagine that the titre is not closely relatable to the clinical action of the preparation but is nothing more than its chemical marker. The clinical results obtained from A will also be repeatable—we have assumed that A is really standardized—and, as frequently happens, will be obviously published. And now imagine that a herbal drug B, nominally corresponding to the same herbal drug A, is developed by another manufacturer, who will market the product and, with the only certainty of being able to use the same botanical species, plant part, and process solvent, will arrive, either with fair means or adulterations, to the same titre of current usage for its marketing. The two products will obviously differ in their nontitrated fraction. It is worth pointing out that the latter may be really enormous in some products. For example, the nontitrated part of St. John's Wort-based herbal drugs accounts for 99.7% of the extract. Now assume that, on the basis of the available literature on the extract really standardized by the manufacturer of A, some clinicians decide to verify the action of product B, nominally equal to A, coming from the same botanical species and same plant part (root, leaves, etc.), obtained using the same solvent, equally titrated and therefore, for the clinician, absolutely comparable to the preparation described in the published papers (actually prepared using A). It is almost sure that, following the use of B, our clinician will not find the same pharmacological/clinical results previously published for product A. This discrepancy is due to an incomplete molecular superimposition between the extract of the publications and the extract actually tested. The reader should not be led to believe that the above example is something exceptional. It is very common. Such highly standardized extracts in all their processing procedures as G115 (*Panax ginseng*), LI160 (*Hypericum perforatum*), or Egb761 (*Ginkgo biloba*) have been the subject of a large number of publications [43–65], probably hardly replicable with the use of common ginseng, St. John's wort, and ginkgo extracts, having the same titration in ginsenosides (7%), hypericin (0.3%), and ginkgoflavonglycosides (24%) plus diterpenes (6%). This can be easily demonstrated through Nuclear Magnetic Resonance (NMR) spectroscopy, an analysis that is anything but simple. The Quali-quantitative and standardization profile analysis of botanical extracts and herbal products is a procedure with two difficult steps, sample preparation and the development of the chemical method for the resolution of analytical peaks. The HPLC quantitative analysis, considered one of the most reliable assays, requires a pure standard of the compound that needs to be quantified. In the absence of precise standards, and for a global assessment of the herbal preparation, the proton NMR analysis will be more useful. In this case the quantitative aspects can be solved simply by resorting to a commercial product preparation which will become the internal reference standard. Use of a deuterated solvent will help trace back the proton spectrum representative of molecular structures (where each peak corresponds to a molecule), and the peak quantification can be made by calculating the relative ratio of the area represented by the proton signals of the assessed compounds with the internal reference standard [66–69]. As can be seen in Figure 1 [70], which shows the proton spectra of two St. John's Wort extracts, the degree of molecular overlap is almost nil, although the extracts are absolutely identical in terms of botanical species, plant parts used, extraction solvent, and final titration. It is logical to think that this poor molecular correspondence can only result in poor reproducibility of the pharmacological and clinical data. Before moving to the following issue of molecular characterization of herbal extracts, I want to inform the reader that in this paragraph I did not take into account the proposed European Medicines Agency (EMEA) categorisation [71] which distinguishes between the concepts of standardization and quantification, where the first corresponds to the adjustment to a defined content of a constituent with known therapeutic activity and the seconds corresponds more simply to the adjustment to a defined range of constituents. European Union has then proposed to classify herbal extracts in three categories: standardized, quantified, and others. This classification of course would affect manufacturers in terms of extract adjustment and stability testing. Anyway the general revision of this classification is still ongoing and not yet definitive, and anyway it is not modifying the message substance of this paragraph.

## 3. The Analytical Problems of a Herbal Drug

**Figure 2:**
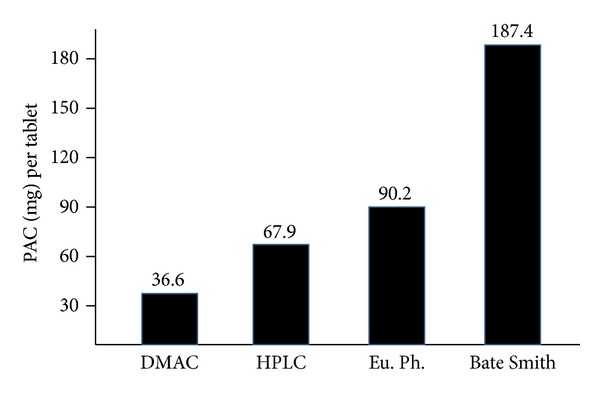
Comparative results of the same cranberry finished product.

As it has been shown, the proton NMR comparison of two herbal extracts developed by two different manufacturers, but obtained from the same botanical species with the same solvent and same chemical titration, seems to demonstrate a really poor qualitative superimposition of their profiles. This problem, as already said, at least in the case of botanical preparations with a well-known correlation between the titrated fraction and the extract action, may be considered a problem of minor importance. Provided that the analytical method used is reproducible and reproduces the reference method. Should this not be the case, our clinician of the above example would again consider 2 different products as being alike. Take the example of* Vaccinium macrocarpon*. For some years now international literature has ascribed the clinical ability of the derivative (juice and/or extract) to prevent relapses in individuals with a diagnosis of recurrent acute cystitis, to the presence of proanthocyanidins of the A2 type (PAC A2 [72]). It is also assumed that the preparation can be effective (i.e., it prevents the subsequent expected relapses when it is administered in a patient with a diagnosis of recurrent cystitis at the time his/her urinoculture being negative) if the latter are contained in a minimum amount of 36 mg per therapeutic dose/day [73]. In this case—that is, when the herbal drug action is correlated with the presence of a titrated fraction—the NMR spectra of 2 products exhibiting qualitative differences (obviously ascribable only to the nontitrated portion) should have effects on the repeatability of the clinical data of minor importance. As a matter of fact it is not just so. As will be demonstrated below, even the nontitrated portions, seemingly useless in an extract, are not really useless. They may be responsible for the processes linked to intestinal absorption of the supposedly active fraction. But this will be dealt with later. Let us go back to the extract part consisting of PAC A2. As clinical efficacy can be obtained through the administration of at least 36 mg/day of PAC A2, our clinician will reason in terms of dose expressed in relation to a titre. If the titre in PAC A2 stated on the pack amounts to 18% of the extract, the clinician will decide to administer 200 mg/day of herbal, as this will correspond to 36 mg/day of active ingredient. Unfortunately, this is not so. The same herbal drug may be titrated through even quite different analytical methods. As shown in Figure 2, a* Vaccinium macrocarpon* extract titrated using 4 different methods (DMAC, HPLC, European Pharmacopeia, and Bate Smith) commonly used to titre PAC A2, gives 4 different results. The question is that the statement about the 36 mg PAC A2 being required for a clinical action applies only to the DMAC method. The other methods do not read the same parameter with the same accuracy [74]. When trying to calibrate the herbal drug to 36 mg/day of PAC A2, detecting the latter using the method of the European Pharmacopeia, the resulting preparations contain a dose that is at least 3 times lower, 12 mg administered per day instead of 36 [75, 76]. The analytical issue, which makes the clinicians think that he is administering a certain dose, while he is actually administering a much lower one, is the second important variable, after the issues of the nonstandardization of botanical preparations, a problem that affects herbal medicine and contributes to the perception of its low clinical efficacy. Let us go back to the example of the clinician. He is sure that he is administering 36 mg/day of PAC A2 and expects the replication of the clinical data described in the literature. But the clinical response is different as the clinician is actually giving the patient 12 mg/die without his knowledge. At the end of the clinical trial, he will conclude that the information on cranberry is not true. Of course, the problem is not just about cranberry. Various herbal drugs are often calibrated by reading an analytical parameter through quite precise analytical methods as those in HPLC [77]. Other manufacturers replicate the numerical titre through much less accurate methods like, for example, spectrophotometric procedures. For example, a ginseng extract HPLC-titrated at 7% in ginsenosides is not equivalent, in the titrated fraction, to a preparation at 7% in ginsenosides obtained through UV reading. Only a 28% UV reading may be theoretically considered quantitatively equivalent to a 7% value obtained through the HPLC method.

## 4. The Interaction with the Organism

As can be evinced from the above considerations, both the problems of phytochemical reproducibility, which could be really solved by an approach aimed at the total standardization of the production processes of herbal drugs, and use of different chemical analysis methods—without any attention being paid to the fact that different methods result in different analytical data in marketed botanicals—lead, or may lead, to nonreproducibility of the expected clinical data. However, nonreproducibility alone does not fully explain the perception of “low pharmacological efficiency” that seems to characterize the herbal products used in food supplements. Of course, the phenomenon is certainly a contributory factor but not enough to explain all of the existing scepticism. This is especially true for the group of preparations (Boswellia, Crataegus, etc.) where the chemical titre is closely linked to the pharmacological action claimed for the whole extract. In order to face this aspect, we need to consider the question of the oral bioavailability of a herbal drug. What happens during the drug transit in the stomach, intestine, and liver? Although it is the same route as that is followed by a synthetic drug, there are unquestionable differences, especially of an evolutionary nature.

## 5. The Poor Oral Bioavailability of Herbal Drugs

**Figure 3:**
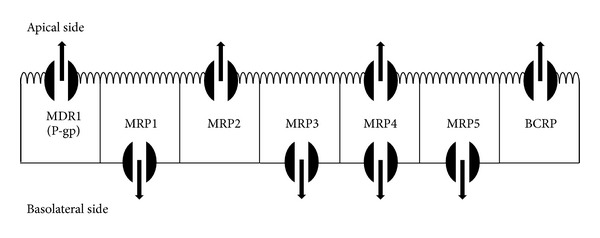
Cellular localization of intestinal ABC transporters (taken and modified from Brand W. et al. Flavonoid-mediated inhibition of intestinal ABC transporters may affect the oral bioavailability of drugs, food-borne toxic compounds, and bioactive ingredients. Biomed Pharmacother. 2006 Nov;60(9):508-19).

A parameter common to most, if not all, of the active botanical ingredients used in the sector of food supplements is their poor oral bioavailability. It is worth noting—although it should not be necessary—that, apart from either extracted or synthetic compounds that do not need to be absorbed to exert their action (e.g., an inhibitor of pancreatic lipase like* Orlistat*, used to reduce hypertriglyceridemia [78]), the pharmacological action of a compound is directly proportional to the percentage capable of reaching the circulating plasma. So the question is how good is the oral bioavailability of herbal compounds? The answer is totally disappointing. Suffice it to think of “famous” substances, easily found in food plants such as curcumin [79], anthocyanins [80], or resveratrol [81], or substances found in medicinal plants like berberine [82], all of which absorbed in lower amounts than 1% (values calculated on the amounts found in plasma following intravenous injection). The reason for such disappointing kinetic data can be explained in several ways, including the possible chemical instability in gastric and/or enteric juices, the degradative metabolism that botanical substances undergo by the action of intestinal flora, and others. A certainly “evolutionary” explanation, usually ignored but nonetheless having a great impact on the kinetic aspects of herbal products, is the role played by cytochromes. Without going into their complex biochemical details, cytochromes belong to 2 categories, Phase I and Phase II enzymes. Phase I enzymes are oxide reductases, primarily responsible for the “demolition” of compounds that, for example, have reached the liver via the portal circuit directly from the intestine. The metabolites resulting from the demolition by oxide reductases are probably more lipophilic than they were before, and, therefore, potentially resorbable by the kidney emunctory. At this point Phase II enzymes take over and combine the metabolite resulting from the action of Phase I oxide reductases with a highly hydrophilic compound (e.g., glucuronic acid) to facilitate its elimination through urine [83]. One should wonder why cytochromes exist. The answer may be enlightening to understand some aspects of the poor pharmacological efficacy of most herbal products. Cytochromes did not exist millions of years ago. Then, with the evolution, they made their appearance about 3 billion years ago and now humans have no less than 110 families [84]. The guide for this evolution was probably the interaction between the plant and the animal, where the former continued to produce new secondary metabolites and the latter protected itself through mutations of the genes encoding for detoxifying enzymes [85]. Secondary metabolites, above all those with chemical aromatic structure, are a group of compounds that plants synthesize starting from shikimic acid. The latter is a reaction intermediate resulting from catabolism of vegetable carbohydrates, which the plant uses as a carbon skeleton to synthesize what might be termed the active ingredients of herbal medicines: anthraquinones, polyphenols, tannins, terpenes, and alkaloids. Things are actually slightly more complex; terpenes derive from a different metabolic intermediate (mevalonic acid), and alkaloids, albeit deriving from shikimic acid, become real alkaloids only after going through structures of branched-chain amino acid. But the previous simplification is acceptable to understand the genesis of secondary metabolites. Defence is essentially the reason for their synthesis by the plant cell. The plant derives secondary metabolites to defend itself, ward off the attacks of animals, and make itself unpalatable. Each of us has similar experiences to report. Just eat an unripe pear and you will understand. What we call unripe and inedible is nothing more than pulp rich in tannins. Its unpalatability prevents animals from feeding on a fruit whose seeds have not yet come to full maturity. When the seeds are ripe, the tannin concentration will decrease, the fruit will be sweeter, and the animals eating it will carry and sow the seeds ingested, but not digested, through the release of their faecal material. The pear concentrates on tannins for defence, to avoid being eaten. This is a simple example that can be easily extended to other experiences. But there are others. Just think of the colours of plants and flowers. They are often chromophoric anthocyanins structures aimed at attracting somehow “fecundating” animals, in particular insects. What we call vegetable active ingredient is actually a toxic metabolite that the plant uses to defend itself or a substance that it uses as a lure. Now the exact correspondence between terms “secondary metabolite” and “vegetable active ingredient” has been clearly identified; let us return to reason about the presence of cytochromes in animals and humans.There must be an evolutionary reason that can explain their existence. It goes without saying. A possible/probable explanation is that the cytochrome system evolved as a mechanism to remove some natural food constituents devoid of any nutritive significance, like polyphenols, terpenes, saponins, and alkaloids [86]. These substances do not provide any nourishment but are found in vegetable food. They do not produce calories. The boxes of food supplements containing herbal products often report this piece of information. In the Nutritional Facts no calories are attributed to these substances. Humans do not break down polyphenols to obtain energy, as they instead do with sugars, proteins, and fats. Polyphenols are useless substances from a nutritive (caloric) point of view. If in an* in vitro* system, which is beyond the issue of absorption and metabolism, we wanted to verify the action of polyphenols; however, we would see that they exhibit some pharmacological properties, for example, a marked antioxidant activity. We are dealing with a group of substances—polyphenols in this case—that does not have any nutritional value but is endowed with pharmacological (antioxidant) properties. What happens then? It happens that, as a form of defence, tissues evolve by eliminating these compounds that it considers useless and dangerous. Tissues and the whole organism behave in the same way with terpenes, saponins, alkaloids, and so forth. All this happens because these compounds, which are now extracted from vegetable matrices, have always been found in plant foods and have been typical of our diet for millions of years. This hypothesis (which is more than an hypothesis) has 2 consequences. The first is that our attempt to concentrate these substances in the so-called herbal food supplements to obtain preparations with pharmacological properties may seem senseless, as these products are “blocked” by man, who has almost been “built” by evolution to block them. This certainly accounts for the particular pharmacological inefficiency of most herbal/food supplements. They are concentrated and administered in individuals who, on the basis of the molecular mould created by these natural substances in foods, have built deactivating (oxide reductases) or conjugating enzymes enhancing their elimination (glucuronidase). A clear and enlightening example is provided by curcumin. Curcumin is poorly absorbed by the intestine, and only a small part of it reaches the liver, where Phase II enzymes are waiting for it. They conjugate it with glucuronic acid, thus reducing it to an almost inexistent free amount in plasma. If we create a direct antagonism to hepatic glucuronidation of curcumin (e.g., by making use of an alkaloid like piperine) glucuronidation of curcumin is reduced, while the plasma level in man, with the same dose of curcumin, increases by about 20 times [87]. The second consequence concerns the aspects of pharmacological interaction with synthetic drugs. When a physician prescribes a medical therapy to a patient, and this decides, maybe without the consent or opinion of the physician, to take a herbal drug (perhaps to weaken the side effects of treatment or for therapeutic reasons that may be different from those intended by the physician but important to the patient), this may lead to possible important interactions. It is the clinician who complains of this. Oddly enough, however, the same clinician who considers the administration of a herbal drug useless thinks that coadministration of the latter with a synthetic pharmacological preparation is dangerous, because he believes that treatment with the useless herbal product may invalidate or otherwise alter the powerful pharmacological treatment he had prescribed. This second issue also sounds inconsistent. Let us assume the theoretical case of the simultaneous administration of 2 compounds: A, a prescribed synthetic drug; B, a self-administered herbal. Both will reach the liver and, therefore, the cytochrome system, at the same time. What will happen? Suppose that compound A is a prodrug and needs to be activated by a cytochrome. The question is which substance exhibits a high degree of molecular affinity, synthetic compound A or herbal compound B? The right answer may be frequently herbal compound B. And it is easy to understand why. On which molecular skeleton did evolution shape a cytochrome, on the carbon skeleton synthesised by a chemist 20 years ago (compound A) or on (for example) the acyl-phluoroglucinol structure typical of hyperforin from St. John's Wort (compound B)? The right answer may be probably on the latter. Here is why compound B will have higher affinity. So on one hand, compound B will be deactivated by the cytochrome, which will thus limit its biological action and, on the other, by occupying the cytochrome with greater efficiency, will limit the activation of the other compound (A), which is needed to be activated to determine a biological effect. There are numberless examples of this [88]. Just think of the risk of unwanted pregnancy in women under contraceptive therapy and consuming St. John's Wort derivatives [89]. The event hypothesised above is all the more evident when we consider substances with a low toxicity profile and, consequently, unable to determine aspects of toxicity in the ingested food. This evolutionary event especially concerns natural substances found in food rather than most venomous substances found in poisonous berries. Interaction with food caused the evolution of the molecular moulds that now limit absorption of botanical compounds, which are still frequently extracted from food matrices. This process did not so much affect those toxic substances that are most concentrated in inedible botanical derivatives and which mammals have learnt, with evolution, to avoid. This consideration may help explain the considerable pharmacological efficacy of vegetable, highly toxic compounds still used successfully in oncology (Vinca derivatives, Yew, and* Camptotheca*), or in other fields (derivatives of Digitalis, Colchicum, and Belladonna), just to mention a few of the best-known examples. This evolutionary process, which led man to a sort of molecular detoxification and today helps to explain, in part, the inefficacy and poor oral bioavailability of herbal drugs, as well as their interactions with synthetic drugs, cannot be solved by simply considering the aspects of liver protection. This is not only due to the fact that cytochromes are widely found in the intestine too, but also because the intestine forms another true and efficient barrier. The intestine is provided with other “detoxification” systems that considerably reduce oral bioavailability of herbals. These systems are the group of ATP-binding cassette transporters (also called ABC proteins, Figure 3), a system of ATP-consummating proteins that extrudes lipophilic compounds permeating the enterocyte into the intestinal lumen, hindering them from reaching the portal circuit and, hence, the liver tissue [90]. The most deeply investigated extrusion pump is multidrug resistance 1 (MDR1) or P-glycoprotein (once also known as Gp-170). It has been the subject of oncological investigation as it is responsible for several chemoresistance events described for antiplastic substances on tumours [91] but is now also the subject of gynaecological investigation as their presence may also explain the resistance to azoles observed with some species of* Candida* [92]. These systems—which have always been present or evolved with time to limit absorption of mycotoxins or other fungal and/or bacterial food-contaminating toxic compounds—interact with different substances of botanical or nonbotanical origin, limiting or otherwise altering their intestinal absorption. An example can be provided by berberine [93]. Up to 90% of the portion administered by mouth is reextruded and thus no longer absorbed, into the intestinal lumen by P-glycoprotein [94]. As with the case of cytochromes, there may be some competition between synthetic compounds and molecules obtained from extraction. A typical example is the alteration of the absorption profile of cyclosporine A (CsA) in the presence of grapefruit polyphenolic extract (or juice). Grapefruit naringenin efficiently interacts with intestinal P-glycoprotein. The latter extrudes part of CsA. When administered together, naringenin will hinder CsA extrusion into the intestinal lumen and enhance its absorption, thus increasing the relevant toxicological hazard [95]. No physician will ever recommend the administration of CsA tablets with a nice glass of grapefruit juice. The presence of intestinal ABC systems does not explain only the low absorption of some herbals or only the possible competition events between synthetic and herbal drugs. It cannot be excluded that their presence undoubtedly generates competition events between different herbal preparations or even between different molecules of the same herbal preparation [96]. This may also explain the kinetic differences observed during purification of a herbal active ingredient starting from an extracted, but less purified, vegetable matrix. An example can be provided by milk thistle. Its extract, known as silymarin, is a mix of flavanolignans consisting almost entirely of silybin, silydianin, and silychristine. Oddly enough, following the administration of equal doses, the bioavailability of pure silybin is much smaller (almost inexistent) than that of silybin administered inside the silymarin matrix [97]. This phenomenon, extendable to most herbal preparations [98], may in some way be attributed to the presence of intestinal ABC systems and their greater or smaller extrusion efficiency in relation to the presence of other compounds. Direct evidence of the above statement can be obtained by verifying the clinical efficacy of a preparation containing berberine alone or berberine and silymarin. Both substances interact with P-glycoprotein, and the performance of berberine is affected negatively by P-glycoprotein-mediated intestinal extrusion. P-glycoprotein antagonists, whether synthetic [99] or natural like silymarin [100], improve both berberine bioavailability and efficacy.

## 6. The Stomach

As described above, the liver and intestine, in relation to the evolutionary theory of cytochromes and ABC systems, help explain some pharmacological/clinical efficiency limitations that are typical of botanical derivatives. The simplified description of these events has been limited to the liver and intestine and has deliberately ignored the issue linked to the role of the blood-brain, placental, or other “barriers,” which rest their functions, or part of their functions, on the same enzyme/protein systems described above and, rationally, modulate the general kinetic aspects of herbal products. Their discussion is beyond the chief arguments of this work and, in any case, they can be considered negligible for the purposes of its general message. On the other hand, mention must be made of the role that the stomach plays or may play in the pharmacological/clinical efficacy of extracts. Making an extract means breaking up the vegetable cell wall and its membranes by means of a solvent, emptying their cell content and retaining the molecular portions, of the cell interior or its membrane, that successfully dissolve in the solvent. If a molecule contained inside a vegetable cell has a strong solubility for a nonpolar solvent like pure ethanol, and extraction is made with hot water, the molecule will not be extracted by the solvent and, since it is not held, will not be part of the extract. The opposite will happen when using pure ethanol. Once the substance has been obtained, it will still be in the extraction solvent, which will have to be removed through desiccation. If the extracted and dried molecule or molecular fraction is food grade, it may be included in a food supplement and administered. It goes without saying that the molecule in question was inside a vegetable cytoplasm with an almost neuter pH value. Following oral administration, the molecule will be located in the stomach, in empty condition, of an individual with a pH close to 1.0–1.5 values. One wonders how many extraction molecules will find themselves “at ease” and therefore stable at such a low pH. As a matter of fact most extracts tend to be manageable in an acid environment and often suffer from an alkaline environment. However, this is not always true. An exception may be that of proteins. Albeit not very common among active ingredients of plant origin, they are part of the phytotherapeutic paraphernalia. Some examples are phaseolamin [101] from* Phaseolus vulgaris*, bromelain [102] from* Ananas comosus*, nattokinase [103] from fermented seeds of* Glycine max,* or again enzymes, such as lipase, [104] obtainable from fermentation of Aspergillus species. As all these proteins have antienzymatic or directly enzymatic properties, they require some “conformational” protection preserving their functional integrity. The low pH of the stomach does not certainly help maintain this functional integrity. Proteins are not the only molecules deserving chemical attention in relation to their transit in the stomach; for example, basic alkaloids undergo deprotonation in the stomach and are partially deactivated and/or slowed down in the subsequent absorption process. When working in the pharmaceutical sector with synthetic molecules exhibiting a partial or total instability in a low-pH environment, the problem is solved through the so-called gastroprotection. This procedure prevents any contact between the active ingredient and gastric acidity, thus eliminating the risk of deactivation. Today, although it is possible to protect botanical extracted active ingredients from gastric juices through excipients (e.g., food grade shellac [105]), this technology is hardly ever used by formulators. Perhaps because too much “pharmaceutical” attention to a food supplement may take it away from those principles of “naturalness” that appeal to the customer and on which “marketing people” rely to promote their sales. Whatever the reason, it can be hypothesized that the lack of attention to the gastric passage reduces the expected clinical actions, inferable from* in vitro* test results, which are not affected by any natural problems linked to the gastric passage.

## 7. Conclusions

In spite of the extremely wide consumption of herbal preparations marketed under the aegis of the rules on food supplements, most physicians and investigators are sceptical about the actual clinical and pharmacological properties of these products. Apart from problems of counterfeiting, adulteration, and sophistication of herbal preparations, the reasons for this scepticism are to be found in the basic aspects of phytotherapy. The chemical complexity of extracts and their difficult molecular characterization (in terms of both their active fractions and apparently useless, but influent, fractions) may explain the poor reproducibility of the results. They also explain the non-confirmation of published data, obtained with well-standardized preparations, which are frequently the subject of pharmaceutical registrations but are hardly replicable in molecular terms. This consideration, absolutely true for those extracts in which the actual correspondence between the molecular contents and the activity of the whole extract has not yet been made somehow clear, is perhaps less important for those extracts in which such a correspondence has been successfully unfolded. However, the latter require an urgent standardization and consolidation of analytical methods to which researchers will have to comply. The nonexplanation as to how different analytical methods may lead investigators to think that 2 preparations are alike, when they are not, feeds the scepticism perceived by clinicians who attempt an approach to the herbal instrument for therapeutic purposes. Even in the case of highly standardized preparations—or preparations that are highly standardized in their titre when the activity is strictly correlated with the fraction—it is better to consider their poor oral bioavailability. The poor oral bioavailability of most herbal drugs, which is due to evolutionary aspects involving at least the cytochrome systems and ABC proteins, helps explain both the reasons for a certain pharmacological inefficiency and a few well-known aspects of pharmacological interaction following the simultaneous administration of synthetic compounds and herbal preparations. Hence the consideration that resort to suitable* ad hoc* antagonists may generate more favourable pharmacokinetic aspects aimed at a more valid therapeutic use of the herbal derivative. As already said, examples of this are provided by antagonisms to hepatic glucuronidation or the use of P-glycoprotein antagonists. No mention has been made of the huge amount of data on the clinical use of “phytosome” forms, which help overcome part of the above kinetic problems and, for herbal remedies susceptible to this complexation, provide decidedly more interesting pharmacokinetic and pharmacodynamic values [106, 107]. The assessment of these approaches (antagonisms and lipidic vehicles) forms another wide phytotherapy topic that is beyond the considerations peculiar to this work. Last, this paper has been written in a deliberately simple language to allow an evaluation by clinicians and investigators who are typically skeptic about use of herbal remedies and, faced with too many technicalities, would have given up reading it.
